# PD-1 Inhibition Enhances Blinatumomab Response in a UCB/PDX Model of Relapsed Pediatric B-Cell Acute Lymphoblastic Leukemia

**DOI:** 10.3389/fonc.2021.642466

**Published:** 2021-04-13

**Authors:** Mark Wunderlich, Nicole Manning, Christina Sexton, Eric O’Brien, Luke Byerly, Cody Stillwell, John P. Perentesis, James C. Mulloy, Benjamin Mizukawa

**Affiliations:** ^1^ Experimental Hematology and Cancer Biology, Cancer and Blood Disease Institute, Cincinnati Children’s Hospital Medical Center, Cincinnati, OH, United States; ^2^ Oncology, Cancer and Blood Disease Institute, Cincinnati Children’s Hospital Medical Center, Cincinnati, OH, United States; ^3^ Department of Pediatrics, University of Cincinnati College of Medicine, Cincinnati, OH, United States

**Keywords:** patient derived xenograft, blinatumomab, pembrolizumab, B-ALL, humanized mice, immune checkpoint inhibition, PD-1, bispecific T-cell engager

## Abstract

Immune therapies such as blinatumomab, CD19-directed bispecific CD3 T-cell Engager (BiTE), have resulted in significant improvements in outcomes for relapsed B-cell acute lymphoblastic leukemia (B-ALL). However, up to half of blinatumomab treated patients do not respond completely or relapse after therapy. As a result, there is a need to identify potential strategies to improve the efficacy of BiTE therapy. The anti-PD-1 antibody pembrolizumab has been shown to successfully activate T cells against a wide range of cancer types. Here, we tested the ability of umbilical cord blood (UCB) reconstituted mice to respond to blinatumomab therapy with or without concurrent pembrolizumab treatment. Humanized mice were engrafted with patient-derived xenograft (PDX) cells derived from pediatric and adolescent/young adult (AYA) B-ALL patients who had either failed to achieve remission with negative minimum residual disease (MRD negative) or experienced a relapse. Mock-treated humanized mice engrafted with PDX cells efficiently developed overt disease within 30 days of engraftment of B-ALL. However, single agent therapy with either blinatumomab or pembrolizumab reduced disease burden in engrafted mice, with some mice observed to be MRD negative after the 28-day treatment course. Combination therapy significantly improved the percentage of MRD negative mice and improved long-term survival and cure rates as compared to mice that were given blinatumomab alone. Importantly, no benefits were observed in treated mice that lacked human immune cell reconstitution. These results indicate that UCB-humanized NRGS mice develop activatable immune function, and UCB-humanized PDX leukemia models can be used in preclinical studies to evaluate specificity, efficacy, and cooperativity of immune therapies in B-ALL.

## Introduction

IL-2Ry immune deficient mice engrafted with human umbilical cord blood (UCB) develop human immune systems with mixed B, T, NK, and myeloid populations. We have previously generated modified NOD/SCID/Gamma (NSG) and NOD/RAG/Gamma (NRG) mouse strains that include transgenic expression of human cytokines SCF, GM-CSF, and IL-3 (NSGS/NRGS) (1, 2). Relative to NSG/NRG, the NSGS/NRGS mice have increased myeloid and NK fractions, more complete B cell differentiation, and faster T cell reconstitution (3, 4). Additionally, both innate and adaptive immune function is also improved as measured by DTH assay and human antigen specific antibody production in response to immunization. NSGS and NRGS mice are also more permissive to human hematopoietic engraftment, particularly in the absence of conditioning (1–3).

B-ALL is the most common hematopoietic malignancy among the pediatric population. While responses to therapy are generally good with survival rates approaching 90% in the pediatric population, a significant number of patients will relapse or be refractory to standard therapy (5). MRD status during and after therapy correlates strongly with relapse free survival and overall survival (6). Recently, new effective therapies for patients in relapse have further improved survival, including the CD19-directed Bi-specific T cell Engager (BiTE), blinatumomab. As a treatment for therapy resistant disease, blinatumomab has been shown to lead to longer relapse free survival compared to historical data using standard chemotherapy approaches in adult patients (7). A group of 20 such patients who received blinatumomab achieved a long-term relapse free survival of 60-65% at 3 years, regardless of HSCT (8). A larger phase 3 trial found that while blinatumomab was more effective than standard chemotherapy for relapsed adult B-ALL in terms of induction of remission and length of overall survival, it was still only effective for fewer than half of patients (9). In a large multi-center trial, blinatumomab therapy lead to increased likelihood of remission and longer survival compared to standard chemotherapy for relapsed adult B-ALL, while the final long-term survival rate was virtually identical (9). This finding suggests that blinatumomab can successfully eliminate leukemia-initiating cells (LICs). In addition, a phase 2 clinical trial showed blinatumomab therapy was effective in adult patients with low level MRD+ disease with significant improvements in EFS, RFS, and OS (10).

Immune checkpoint inhibitors (CPI) targeting ligand-receptor interactions that suppress immune effector cell activation have entered wide use in cancer therapy. Pembrolizumab, an anti-PD-1 blocking antibody, is one such CPI that stimulates immune activity against a wide range of malignancies. It prevents ligation to PD-L1, a surface ligand which is frequently found upregulated on cancer cells and provides inhibitory signaling to the T cell as an immune escape mechanism. BM samples from B-ALL patients at both diagnosis and relapse contain T cells with increased PD-1 expression, relative to healthy controls or patients in remission (11–13). Additionally, increased PD-L1 expression has been noted on relapsed B-ALL with increased levels on blinatumomab non-responder blasts, compared to responders (12, 14). Addition of blinatumomab to an ex vivo co-culture of patient blasts and a B-ALL cell line resulted in a further upregulation of PD-1, but not when healthy donor lymphocytes were used in place of leukemic blasts, indicating that this phenomenon was mediated by the leukemia cells. Combination of pembrolizumab with blinatumomab resulted in a temporary response in one patient that was previously blinatumomab resistant, although it is unclear whether this result could have been realized with pembrolizumab alone since donor T lymphocytes were also infused at the same time (12).

Recently, several models have been developed to study immune therapy in a xenograft setting. NSG mice engrafted with PBMNCs demonstrated efficacy of a novel dual CD3XPDL1 BiTE against a xenografted melanoma cell line (15). However, PBMNC models are problematic due to the rapid onset of GVHD. Double knockout of MHC class I and II in the NOG strain allowed GVHD-free engraftment of PBMNCs which responded to a lab made PD-1 antibody and slowed the growth of two partially MHC matched human cell lines *in vivo (* 16). Also, NSG mice engrafted with fetal liver HSCs generated a more complete human immune system which was able to slow tumor growth of HCC PDX tumors upon either pembrolizumab or ipilimumab treatment (17). Finally, UCB reconstituted NSG mice also generated pembrolizumab-responsive human immune systems that successfully slowed the growth of a variety of solid tumor PDX and CDX models (18). UCB based models have the benefits of relying on a cheap and readily available source of HSCs that is not labor intensive and has limited risk of acute GVHD.

Here, we developed a tractable system to test immunotherapy in UCB-reconstituted NSGS and NRGS mice engrafted with B-ALL PDX models. Mice treated with either blinatumomab or pembrolizumab were partially protected from disease at day 30 by flow MRD analysis. The combination of blinatumomab and pembrolizumab resulted in fewer mice with detectable MRD and improved survival. These results demonstrate the utility of dual UCB/PDX models for examining novel combinations of immune therapies *in vivo* that may improve response rates of relapsed and refractory pediatric B-ALL.

## Methods

### UCB Humanization

UCB was obtained from the Translational Trials Development Support Laboratory of CCHMC. Units were RBC depleted by hetastarch sedimentation and unselected WBCs were viably frozen in IMDM/50%Hespan/25%BSA/5%DMSO until needed. At thaw, cells were mixed with OKT3 and intravenously (IV) injected into busulfan conditioned mice (2). PB was monitored for the appearance of human CD45+CD3+ T cells by flow cytometry.

### PDX Models

Previously generated PDX models were obtained from the Pediatric Leukemia Avatar Program of the Cancer & Blood Diseases Institute (CBDI) at Cincinnati Children’s Hospital Medical Center (CCHMC). Original patient material was collected under IRB approved protocols. Viably frozen primary and secondary spleen preparations were used to initiate B-ALL in the cohorts described here.

### Antibody Treatments

Discarded residual aliquots of blinatumomab and pembrolizumab were obtained from the CCHMC pharmacy. Blinatumomab (Blincyto, 12.5ug/mL) was diluted to 0.25ug/mL in sterile PBS/3%FBS with antibiotics. Each dose consisted of a 250uL ip injection (approximately 2.0-2.5ug/kg). Pembrolizumab (Keytruda, 25mg/mL) was diluted to 1mg/mL with PBS/3% FBS and antibiotics. Each dose was a 300uL ip injection (approximately 10mg/kg). Antibody treatments started the day of B-ALL engraftment. Blinatumomab injections were repeated daily for up to 4 weeks and pembrolizumab was given once or twice on days 1 and 15 (see **Table 1** for specific details).

**Table 1:** PDX model summary.

Experiment	PDX ID	Patient History	Cytogenetics	Blina Response	Mouse Strain	UCB Transplant	PDX Cell#	PDX Treatments
Expt#1	ALL #1	Infant bi-lineage leukemia; relapse w/B-ALL post-HSCT	t(6;11)	PD; CD19+	NSGS	8.0M WBCs w/OKT3, IV	3.0M	Blina 2X per day ip (4 weeks), Pembro 1^st^ and 3^rd^ Monday
Expt#2	ALL #2	B-ALL; early marrow relapse	t(1;19)	PD; CD19+	NSGS	7.0M WBCs w/OKT3, IV	2.5M	Blina 2X per day ip (2 weeks), Pembro 1^st^ Monday
Expt#3	ALL #3	Ph+(T315I) B-ALL, MRD negative post-Blina; relapse post-HSCT	t(9;22)	CR; later CD19+ relapse post-HSCT	NRGS	8.0M WBCs w/OKT3, IV	1.0M	Blina 1X per day ip (4 weeks), Pembro 1^st^ and 3^rd^ Monday
Expt#4	ALL #4	Infant B-ALL; primary refractory disease	t(4;11)	PD; CD19-	NRGS	6.3M WBCs w/OKT3, IV	3.0M	Blina 1X per day ip (4 weeks), Pembro 1^st^ and 3^rd^ Monday

PDX, patient-derived xenograft; ALL, acute lymphoblastic leukemia; HSCT, hematopoietic stem cell transplant; Ph, Philadelphia chromosome; Blina, blinatumomab; PD, progressive disease; CR, complete remission; WBC, white blood cell; UCB, umbilical cord blood; Pembro, pembrolizumab.

### Transfusion

Some mice were given transfusions to relieve anemia that has been described to occur in NSGS and NRGS mice with human immune reconstitution (19). Donor mice were bled from the tail into a 1.5mL tube containing 100uL heparin (Sigma Aldrich #2106 - dissolved in 1mL PBS). 400uL PB was added to each tube. This sample was immediately iv injected into 2 recipient mice (250uL each).

### Statistics

Mann-Whitney and paired tTests were performed with Prism 8 software (GraphPad). Log rank test was performed online at http://bioinf.wehi.edu.au/software/russell/logrank/. Significance was set to p<0.05.

## Results

### Pembrolizumab in Combination With Blinatumomab Improves Clearance of B-ALL in PDX Mice

**Figure 1:**
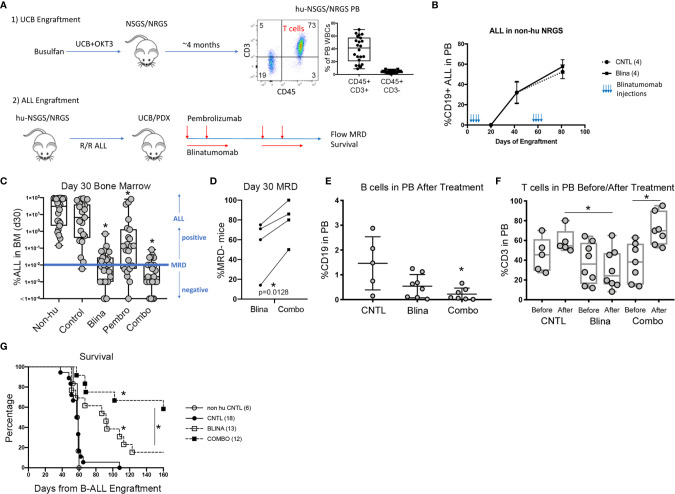
Modeling combined blinatumomab (Blina) and PD-1 inhibition with pembrolizumab (Pembro) in humanized mice. **(A)** Schematic outlining the experimental setup. **(B)** Non-humanized mice were treated with Blina or control (CNTL) immediately after engraftment (2 weeks, beginning on day 1) with B-ALL and were re-treated after disease was readily detectable in PB (beginning on day 48). **(C)** B-ALL engraftment (from 4 unique PDX models) was detected in the bone marrow of mice 30 days after therapy was initiated. Treatment was started on day 1 of B-ALL injection according to the schedules in **Table 1**. *p < 0.05 by Mann-Whitney test. **(D)** Proportion of mice with lower than 0.01% B-ALL in the marrow (MRD-) at day 30 for each of the 4 samples in Blina or combo groups. *p = 0.0128 by paired tTest. **(E)** Flow cytometry measurement of B cells in the PB of mice after therapy. *p < 0.05 by Mann-Whitney test. **(F)** Flow cytometry measurement of T cells in the PB of mice before and after therapy. *p < 0.05 by Mann-Whitney test. **(G)** Survival of treated mice correlates with MRD flow detection. *p < 0.05 by log rank test.

To test the suitability of humanized mice to respond to immune modulatory therapies for B-ALL, we first generated UCB engrafted mice with detectable T cells in circulation (**Figure 1A**). After approximately 4 months, the majority of human CD45+ cells were found to be human CD3+ T cells. One of several B-ALL PDX models (**Table 1**) was then engrafted prior to treatment with blinatumomab with or without pembrolizumab.

We began by testing if blinatumomab would have any direct effects on engraftment or expansion of B-ALL in mice without human immune reconstitution. Mouse macrophages could potentially eliminate or reduce leukemic burden if they successfully recognize blinatumomab-labeled cells. Also, some antibodies may directly affect the target cells. Importantly, we observed no delay in development or reduction of established human B-ALL in the PB of engrafted mice that received blinatumomab in absence of human immune reconstitution (**Figure 1B**).

Next, we engrafted humanized mice with a B-ALL PDX sample and initiated treatment with either blinatumomab, pembrolizumab, or the combination (**Figure 1C**). Non-humanized control (Non-hu CNTL) mice developed robust disease within 30 days as measured by bone marrow aspiration and disease specific flow cytometry. In humanized mice that did not receive either specific antibody, a small but inconsistent decrease in disease levels was seen. Blinatumomab treated mice had a significantly lower disease level in the BM while pembrolizumab treatment also led to a significant reduction in disease, although to a lesser degree. Combination therapy resulted in the greatest reduction in detectable disease and more mice with MRD negative bone marrow (fewer than 0.01% leukemic blasts, the clinical cutoff for flow cytometry based MRD assessment). These results were consistent for 4 separate PDX samples derived from relapsed B-ALL (**Figure 1C** and **Table 1**) with combination treatment leading to a significantly higher proportion of MRD negative mice (paired tTest, p=0.0128, **Figure 1D** and **Table 2**).

**Table 2:** Fraction of MRD- mice at day 30.

Treatment Group	Expt #1	Expt #2	Expt #3	Expt #4	Total	Percentage
Non-hu CNTL	0 of 6	0 of 7	0 of 5	0 of 5	0 of 23	0%
CNTL	1 of 14	1 of 6	0 of 5	0 of 6	2 of 31	6%
Blinatumomab	5 of 7	3 of 4	3 of 5	1 of 7	12 of 23	52%
Pembrolizumab	1 of 6	3 of 4	3 of 5	0 of 7	7 of 22	32%
Combination	6 of 7	5 of 5	4 of 5	3 of 6	18 of 23	78%*

*Paired tTest, Blinatumomab vs. Combination = 0.0128.

### Pembrolizumab Reverses T Cell Lymphopenia Induced by Blinatumomab

A significant drop in peripheral B cells was observed when we measured CD19+ B cells in the PB of humanized mice with B-ALL engraftment after treatment with blinatumomab, reminiscent of the B cell aplasia observed in patients (**Figure 1E**). This decrease was more dramatic in the combination cohort, possibly indicating increased killing of both normal and malignant B cells. Analysis of CD3+ T cell numbers in the PB before and after therapy showed peripheral T cells increased slightly in control untreated mice over time, which is expected since T cells gradually increase as a percentage of WBCs in xenograft PB over time (**Figure 1F**). In contrast, peripheral T cells dropped in most blinatumomab treated mice. However, the addition of pembrolizumab reversed this effect and combination treated mice had increased T cells after treatment, which might indicate a mechanism for increased efficacy.

### Combination Therapy Increases Survival of UCB-Humanized Mice With B-ALL Engraftment

Following MRD assessment, we monitored mice for long term survival. Untreated humanized mice succumbed to B-ALL between 40 and 60 days after engraftment (**Figure 1G**). Most of the blinatumomab treated mice developed fatal leukemia with delayed kinetics, however there were 2 long-term survivors. A significantly higher proportion of combination treated mice survived until the end of the experiment. As a functional test for residual leukemia initiating cells (LICs), we harvested BM from these surviving mice and transplanted whole marrow into busulfan conditioned secondary hosts. None of the flow cytometry-based MRD negative samples generated leukemia indicating a lack of transfer of undetected residual disease from the primary mouse. All mice remained disease free in the secondary graft at 120 days suggesting the survival benefit seen in the primary cohort is durable and represents clearance of leukemia-initiating cells.

## Discussion

Here we have established a model system to examine immune therapies in the context of a humanized mouse reconstituted with UCB hematopoietic stem cells. The method relies on a readily available source of stem cells and avoids GVHD and labor-intensive steps of other models. Our model of early treatment with blinatumomab after engraftment mimics several completed and ongoing clinical trials in which blinatumomab has been used against residual post chemotherapy MRD (7, 10, 20). One question that remains unanswered is if blinatumomab could be used concurrently or consecutively with conventional chemotherapy, and how the lymphotoxic effects of chemotherapy impact efficacy of blinatumomab (21). We have recently optimized conditions for modeling standard 4-drug ALL induction protocols in xenografts which could be used to investigate this possibility (22).

One common toxicity with blinatumomab therapy in cases of overt disease is the development of cytokine release syndrome (CRS) which appears to progress to hemophagocytic lymphohistiocytosis or macrophage activation syndrome (MAS) (23, 24). One case report reported on the use of tocilizumab to target the IL-6R in a patient experiencing severe CRS-induced MAS (25). While there was a rapid clinical improvement of CRS symptoms, it remains unclear if targeting IL-6 signaling interferes with the activity of blinatumomab or whether the two therapies can be delivered simultaneously without affecting the anti-leukemic effect. The UCB reconstituted NSGS/NRGS mice we used in this study do in fact develop a progressive, myeloid driven MAS over time that responds to either gemtuzumab ozogamicin or tocilizumab (19). It would be interesting to test whether blinatumomab therapy would induce CRS and exacerbate MAS in mice with higher initial levels of B-ALL. If so, our model could evaluate the effects of tocilizumab and other potential therapies that might treat CRS/MAS while preserving the efficacy of blinatumomab.

The drop in PB B and T cells after blinatumomab treatment is consistent with long term elimination of B cells and a transient drop in PB T cells observed in patients (26). However, in patients, T cell levels reached a nadir after 1-2 days and recovered to normal levels within 8-10 days before expansion over baseline (particularly for CD8+ T cells) (20) which was observed for the duration of the 28-day cycle. A drop in T cells was observed in the beginning of each cycle. Levels of T cell expansion during blinatumomab treatment has been shown to correlate with long term survival in patients that achieve MRD negative status (27). We are unable to replicate the slow constant infusion of blinatumomab and instead must rely on repeated bolus injections which may result in increased elimination of T cells and explain the lack of expansion. We did however observe T cell expansion after combination therapy, indicating that immune checkpoint inhibition may serve to counteract the T cell lymphopenia associated with blinatumomab. It remains unclear whether this quantitative increase in PB T cell numbers is directly related to improved efficacy.

The B-ALL PDX models used in these experiments represent cases of relapsed or persistent residual disease for which blinatumomab has established clinical indication. Two of our PDX ALL models (#1 and #2) were derived from patient samples representing relapsed disease that was unsuccessfully salvaged with blinatumomab. In both cases, the disease that progressed during blinatumomab treatment remained CD19+, suggesting immune escape or anergy. PDX model ALL#3 was derived from a patient who achieved an MRD negative remission after blinatumomab and subsequently underwent allogeneic stem cell transplant, only to relapse post-transplant with CD19+ disease. This illustrates the great need for strategies to enhance the efficacy of immunotherapies. B-ALL PDX models in human immune cell-reconstituted mice provide a valuable resource for development and validation of those strategies.

In these experiments, samples from patients that were refractory to blinatumomab in the clinical setting nevertheless showed some response in the PDX experiments. This may be due to the use of a healthy UCB-derived immune cell repertoire, as opposed to the endogenous immune cells that may be compromised in number or function in patients heavily pretreated with cytotoxic chemotherapy. This PDX system provides one avenue to investigate whether CPI failure results from leukemia-intrinsic factors, or from defects in the immune milieu.

A number of clinical trials have been initiated to assess the efficacy of combined blinatumomab with CPI, such as pembrolizumab or nivolumab, in the treatment of B-ALL. Preliminary findings in a phase I clinical trial of blinatumomab and nivolumab in adults with relapsed/refractory (R/R) CD19 positive B-ALL demonstrated favorable safety profile and clearance of MRD in this heavily pretreated cohort (28). A similar study will examine this approach in pediatric and adolescent/young adult (AYA) patients with R/R CD19 positive B-ALL (NCT04546399). Combinations of immune therapies may have dramatically different efficacies depending on timing and sequence as has been shown in a mouse model of breast cancer combining anti-PD-1 with anti-OX40 antibodies (29). Similarly, the combination of blinatumomab and nivolumab is being investigated in clinical trials to determine whether CTLA-4 inhibition with ipilimumab can further augment the immune response to eliminate CD19 positive B-ALL (NCT02879695). The humanized models of immune therapy in leukemia PDX mice presented here could provide an important preclinical testing platform for examining the efficacy of such combinations and their mechanisms of response, resistance, and toxicity.

## Data Availability Statement

The raw data supporting the conclusions of this article will be made available by the authors, without undue reservation.

## Ethics Statement

The studies involving human participants were reviewed and approved by Cincinnati Children’s Hospital Medical Center Institutional Review Board. Written informed consent from the participants’ legal guardian/next of kin was not required to participate in this study in accordance with the national legislation and the institutional requirements. The animal study was reviewed and approved by Cincinnati Children’s Hospital Medical Center Institutional Animal Care and Use Committee.

## Author Contributions

Study Design: MW, JM, and BM. Experimentation and Data Collection: MW, NM, CSe, EO’B, LB, and CSt. Data Interpretation: MW, JM, and BM. Drafting Manuscript: MW. Editing and Revision: JM and BM. Samples and Reagents: EO’B, LB, JP, and BM. Funding: MW, JP, and JM. All authors contributed to the article and approved the submitted version.

## Funding

This work was supported by NIH/NCI R50 CA211404 (MW), NIH/NCI R01 CA204895 (JM) NIH/NCI R01 CA215504 (JM), and a CCHMC ARC award (JP and JM). Flow cytometry was supported in part by NIH grant P30AR070549.

## Conflict of Interest

The authors declare that the research was conducted in the absence of any commercial or financial relationships that could be construed as a potential conflict of interest.
